# Evaluating new simplified assays for harm reduction from methanol poisoning using chromotropic acid kits: An analytical study on Indian and Iranian alcoholic beverages

**DOI:** 10.3389/fpubh.2022.983663

**Published:** 2022-11-03

**Authors:** Ali Rafizadeh, Ashish Bhalla, Neha Sharma, Kuldeep Kumar, Nasim Zamani, Rebecca McDonald, Darren M. Roberts, Hossein Hassanian-Moghaddam

**Affiliations:** ^1^Department of Nursing and Midwifery, Islamic Azad University, Rasht, Iran; ^2^Department of Internal Medicine-Toxicology, Postgraduate Institute of Medical Education and Research, Chandigarh, India; ^3^Department of Biophysics, PGIMER, Chandigarh, India; ^4^Department of Clinical Toxicology, Loghman Hakim Hospital, Shahid Beheshti University of Medical Sciences, Tehran, Iran; ^5^Social Determinants of Health Research Center, Shahid Beheshti University of Medical Sciences, Tehran, Iran; ^6^Norwegian Centre for Addiction Research (SERAF), University of Oslo, Oslo, Norway; ^7^Drug Health Services, Royal Prince Alfred Hospital, Camperdown, NSW, Australia; ^8^St Vincent's Clinical School, University of New South Wales, Darlinghurst, NSW, Australia

**Keywords:** methanol toxicity, adulterated alcohols, alcoholic drinks consumption, prevention, methyl alcohol

## Abstract

**Background:**

Ingestion of methanol can result in severe irreversible morbidity, and death. Simple and easy methods to detect methanol and other hazardous additives prior to consumption can prevent fatalities. This form of harm reduction is analogous to the widely practiced “pill testing” of recreational drugs in various countries. We aimed to evaluate the performance of two qualitative and quantitative kits to simultaneously identify the presence of methanol and formaldehyde in alcoholic beverages, and compare this to the standard gas chromatographic (GC) method.

**Methods:**

Two-hundred samples of Indian and Iranian alcoholic drinks were examined by two new qualitative and quantitative chemical kits designed based on a modified chromotropic acid (CA) method, as well as a gold standard GC method.

**Results:**

Methanol levels were similar when evaluated by GC and quantitative method (*Z* = – 0.328, *p* = 0.743). The 75th percentile of methanol level detection was 4,290 mg L^−1^ (range; 0–83,132) using GC compared to that of 4,671 mg L^−1^ (range; 0–84,960) using the qualitative kit (predefined color intensity reflecting the methanol/ethanol ratio). The quantitative kit was able to detect all methanol-contaminated and non-contaminated samples (110 and 60 cases, respectively: 100% sensitivity). In 25 samples, GC analysis showed no methanol; but the qualitative kit detected possible toxic substances. Formaldehyde measurement by UV/Vis analysis showed the presence of formaldehyde in 23 samples (92%) with a median 912 [IQR 249, 2,109; range 112–2,742] mg L^−1^.

**Conclusion:**

Methanol and formaldehyde can be easily detected using these simple CA chemical kits. Qualitative positive results may indicate the risk of poisoning if the beverage is consumed. CA kits can be used in community setting by public health units and community organizations to monitor for methanol contamination and inform a public health response to reduce methanol-related harms to the public.

## Introduction

Although ethanol is the most important chemical of alcoholic drinks, compounds including acids, aldehydes, ketones, and esters always exist in alcoholic beverages even in small quantities and can result in the special features of the product including its color, smell, and taste (1–3). Methanol is the hazardous chemical with the second highest allowable concentration in alcoholic beverages. It is generally produced because of decomposition of pectin during the process of sweet fruits fermentation (1, 4, 5). Ingestion of methanol is a major cause of mortality and morbidity worldwide (4–9). Methanol exposures largely occur due to its contamination or substitution into beverages intended for human consumption. Home-made beverages containing methanol were recently responsible for several methanol outbreaks in Iran and India (10–13).

However, the potential toxicity of methanol depends on the ethanol to methanol ratio. According to the European standards, the methanol content of alcoholic drinks should not exceed the equivalent of 10 grams in one liter (g L^−1^) of absolute ethanol or 4,000 mg in one liter (mg L^−1^) of a 40% v/v alcoholic drink. The ethanol concentration in products sold as beverages vary from 4 to 60% volume/volume [v/v]. Because ethanol is the antidote to methanol, high concentrations of methanol are required to cause methanol poisoning in high-content ethanol beverages (5, 8, 14).

Very low quantities of formaldehyde are also expected in alcoholic beverages (15). Currently, there is no systematic data about the formaldehyde content of the alcoholic beverages. However, formaldehyde was categorized as a carcinogenic to humans by the International Agency for Research on Cancer (IARC). The United States Environmental Protection Agency recommended daily intake of formaldehyde should not exceed 0.2 mg_/_kg^−1^ of the body weight while WHO set it at 0.15 mg kg^−1^ of the body weight (16). So, the methanol and formaldehyde concentration of alcoholic drinks is of concern in the quality control process.

Several advanced methods including high-performance liquid chromatography (HPLC), selective flow injection, enzymatic method, Fourier-transform infrared spectroscopy (FT-IR), gas chromatography-mass spectrometry (GC–MS), and gas chromatography (GC; the usual gold standard) are used to evaluate methanol and formaldehyde contents of different samples (15, 17, 18). Most of these methods require sample pretreatment, expensive equipment, long operation time, and high technical knowledge/experience, each of which are barriers to incorporating these assays into routine processes in laboratories of resource poor and developing countries (18, 19). Additionally, formaldehyde and methanol cannot be simultaneously evaluated during a typical GC test which is commonly performed to identify or measure the methanol concentration of various samples (20). Due to physicochemical differences between alcohols and aldehydes it is not possible to measure both methanol and formaldehyde in the same analysis. Thus, separate analytical protocols must be implemented to determine the formaldehyde content using GC. Unlike methanol, many chemical methods can be used to determine formaldehyde including chromotropic acid (CA) which is the most efficient. This old, sensitive and specific reference colorimetric method was recommended for measuring a wide range (20–4,000 mg L^−1^) of formaldehyde and its releasing compounds (such as methanol) by National Institute for Occupational Safety and Health (NIOSH) and Association of Official Analytical Chemists (AOAC) (21, 22). Using this method, the simultaneous measurement of both formaldehyde and methanol is possible (20). However, using high volumes of hot concentrated sulfuric acid (potentially high risk) and application difficulties (long operation time and detailed processes) prevents its routine use in laboratories (4, 5). Therefore, developing safer and easier methods are encouraged in order to identify products intended for consumption which contain toxic methanol concentrations in countries such as Iran and India.

It is anticipated that if this testing could be introduced into communities consuming these beverages that it would be an effective harm minimization strategy. This practice of ‘drug checking' has been implemented in some countries for the testing of recreational substances (23–26). Various testing methods have been employed to identify unexpected, potentially toxic substance, prior to intake (27, 28). This data can inform individuals regarding potential risks of the substance, prior to use.

The main goal of this study was to identify an accessible and practical method for evaluating Iranian and Indian alcoholic beverages for toxic substances. The study aimed to do this by evaluating the performance of two qualitative and quantitative kits to simultaneously determine the risk of methanol and formaldehyde toxicity in almost 200 Iranian and Indian alcoholic samples, and compare the results with those from the standard GC analytical method.

## Methods

Two-hundred samples of Indian and Iranian alcoholic drinks (described below) were examined by newly developed qualitative and quantitative chemical kits based on a modified CA method, and GC (the gold standard) in India. The results were then compared. Different technicians performed each type of assay, and each technicians was blinded to the results obtained by the others. Confirmatory tests were performed in different labs in Iran.

Methanol and ethanol contents of all samples were firstly determined by the GC method. The qualitative kit was used to detect the potential for methanol toxicity in the samples on the basis of the methanol to ethanol ratio, and the exact methanol content was determined by a quantitative kit. According to kits' brochures, methanol and formaldehyde (if present) concentrations were simultaneously evaluated.

### Instruments

The GC instrument used was a YL 6,100 model of Yanglin (South Korea). The GC system was equipped with a flame ionization detector (FID) and Tr2b-5 column. The length and inner diameter of the capillary column was 30 m and 0.53 mm, respectively. Helium (flow rate = 4 mL min^−1^) was used as a carrier gas for methanol separation. Two spectrometers (Jenway single beam 6,405 [UK] and PerkinElmer double beams Lambda UV/VIS scanner [USA]) were used to measure methanol and formaldehyde content of the samples, respectively.

### Chemicals

The reference chemicals ethanol, methanol, formaldehyde, 1-propanol, chromotropic acid, and concentrated sulfuric acid were purchased from Merck (Darmstadt, Germany) and used without further purification. Deionized double distilled water (D.W.) was used to prepare standard and control solutions. The qualitative and quantitative kits (Arya Mabna Tashkhis Co., Tehran, Iran) were provided by this company to perform the tests.

### CA method mechanism

In the CA method, methanol is oxidized to formaldehyde, and then to formic acid using potassium permanganate in acidic media. The dark color of the solution (violet) is then faded by sodium hydrogen sulfite (converts violet Mn^7+^ to colorless Mn^2+^). Then, the formic acid is reduced to formaldehyde which reacts with CA in the presence of heated concentrated sulfuric acid which produces a violet complex. The intensity of the violet color is proportional to the methanol concentration (29, 30).

### Qualitative detection of methanol and formaldehyde toxicity risk

**Figure 1:**
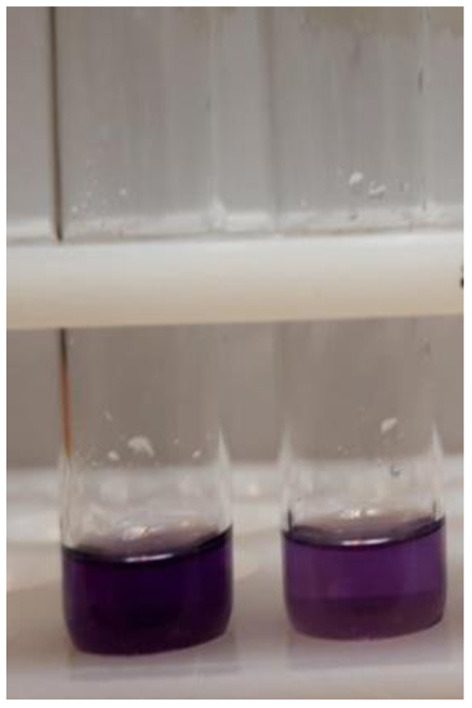
Two positive results.

The qualitative kit contained five reactants (A, B, C, D, and E), a calibrated color band for interpreting the results, and a brochure. The utility of this kit was demonstrated in a previous study (2). This kit evaluates methanol intoxication risk by detection of the methanol to ethanol ratio. To detect the risk of methanol and formaldehyde intoxication of samples by this kit, 50 μL of each sample was poured into a clean test tube with 50 μL of A and B reactants (sulfuric acid and potassium permanganate solutions, respectively) and shaken. Three mins ater, 50 μL of C (sodium hydrogen sulfite) reactant was added to the test tube to fade the color. Finally, 50 μL of D (CA) and 1 mL of E (concentrated sulfuric acid) reactants were added to the test tube and mixed. Up to five min later, at room temperature, the result was observed by comparison of the color with the reference band as follow:

Positive or high-risk: if the obtained result was darker than the reference band indicating methanol to ethanol ratio of the sample to be more than the EU standard (hazardous). Figure 1 is an example of a positive reaction.

**Figure 2:**
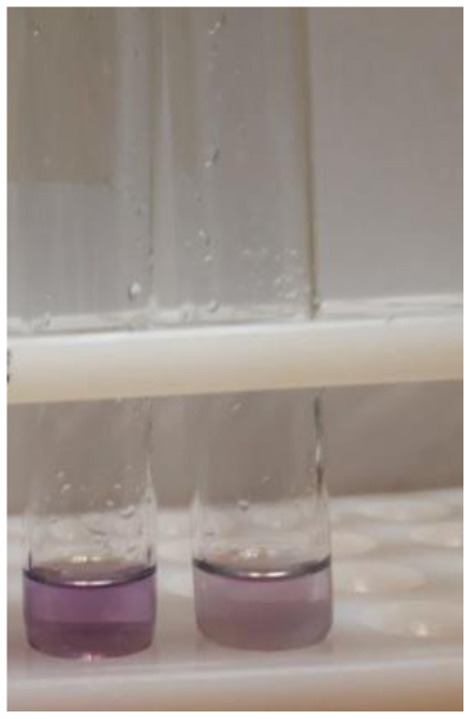
Two negative results.

Negative or low-risk: if the solution remained colorless or the obtained color was lighter than the reference band which indicated methanol to ethanol ratio to be equal to or less than the EU standard (safe). Figure 2 is an example of a negative reaction.

### Quantitative measurement of methanol by kit

The quantitative kit contains five reactants (A, B, C, D, and E), five standards with 0, 12.5, 25, 50, and 100 mg L^−1^ values of methanol, and a brochure with instructions. This kit was used to determine the exact methanol content of the samples by colorimetric method. The utility of this kit was demonstrated in a previous study (5). The Limit of Quantification (LOQ) of this kit was 1,500 mg L^−1^. To perform the tests by this kit, all samples and controls should be diluted to 1:100 by D.W. and then, 50 μL of all standards, diluted controls and samples were poured into separate previously labeled test tubes with 50 μL of A and 100 μL of B reactants (sulfuric acid and potassium permanganate solutions, respectively), and shaken. After 15 min, 50 μL of C reactant (sodium hydrogen sulfite) was added to the test tubes to fade the color by hard shaking. Finally, 50 μL and 1 mL of D (CA) and 1 mL of E (concentrated sulfuric acid) were respectively added to the test tubes and mixed. After cooling test tubes at room temperature, their absorbance was read at 575 nm in contrast to D.W. and their methanol content was determined by comparing the results with the standard curve and multiplying the obtained result by the dilution factor (or 100).

### Quantitative measurement of formaldehyde

For measurement of formaldehyde in the samples were positive by qualitative assays and negative by GC method (false positive results of qualitative methanol kit), a modified NIOSH procedure of chromotropic acid reference method was used. A 5% w/v aqueous solution of chromotropic acid was prepared to determine the formaldehyde content of the samples by colorimetric method. Three control solutions were also prepared with 500, 1,000, and 2,000 mg L^−1^ of formaldehyde in aqueous ethanol solution with 40% v/v alcoholic strength to be used in the colorimetric method. To measure the formaldehyde content, all samples and controls were diluted with a 1:10 ratio with D.W. To perform the tests, 100 μL of all standards, diluted controls and samples (with 1:10 ratio) were poured into separate previously labeled test tubes with 50 μL of prepared aqueous solution of CA and 1 ml of concentrated sulfuric acid and mixed by hard shaking. After the test tubes were cooled down at room temperature, their absorbance was scanned at 565–585 nm ranges in contrast to D.W. and their average was calculated in comparison to the standard curve by multiplying the obtained result of formaldehyde content by the dilution factor (or 10).

### Quantitative measurement of methanol and ethanol contents by GC

To evaluate the methanol and ethanol contents of the samples using GC, five mixed standard solutions with 800–12,800 mg L^−1^ concentrations were prepared in D.W. Three control solutions were also prepared with 2,000, 4,000, and 8,000 mg L^−1^ of methanol in aqueous ethanol solution with 40% v/v alcoholic strength to be used in both GC and quantitative methods kits. One-propanol aqueous solution was then prepared to be used as the internal standard in GC method. The exact ethanol and methanol contents of the samples were initially determined by the GC method by a 10 μL Hamilton Syringe. For this purpose, all standards, controls, and samples were directly injected into the GC system. To increase the accuracy of the results, a prepared 1-propanol aqueous solution was added to all used standards, controls and samples as internal standard to reach 200 mg L^−1^ concentration.

To determine ethanol and methanol contents of the samples by GC method, 2 μL of all prepared standards and samples were directly injected into the GC system with column temperature pre-incubated at 50°C for a minute followed by 10°C min^−1^ increase to 80°C. The oven, injector and detector temperatures were fixed at 80, 240, and 280°C, respectively. The total run time of each test was 10 min.

### Samples

All 100 Indian samples were purchased from official local stores of Chandigarh, India.

Due to the religious prohibition of consumption of alcoholic drinks in Iran and limited access, only 25 samples were provided from the Iranian local black markets. They were either smuggled (illegally imported) into the country, or domestically prepared. The homemade samples were poured in non-standard plastic bottles with no label. Judging the quality of the samples was impossible, but estimated based on the price, declaration of the vendor, taste, and smell of the beverage.

The remaining Iranian samples were made from five methanol-free pure ethanol drinks by adding methanol to form known concentrations. This process also explored the matrix effects of these products (especially, ethanol concentration) on the performance of the kit (add-found technique) (31, 32).

### Evaluation and interpretation of measurements

While the methanol and formaldehyde contents of the samples were evaluated by three different methods (GC/two chemical methods), the ethanol level was only determined by GC.

Comparison of the quantitative kit results to GC was done by calculation of risk of poisoning based on the permitted dose of methanol in alcoholic drinks (European standard or 4,000 mg L^−1^ in 40% alcohol) considering limit of Quantification (LOQ) of the kit (1,500 mg L^−1^). In other words, the 1:100 ratio of methanol to ethanol (for every 1% v/v or 10,000 mg L^−1^ of absolute ethanol, 100 mg L^−1^ of methanol is only acceptable) was applied for this purpose.

### Statistical analysis

Statistical package for social sciences (SPSS) version 24 (IBM Corporations, Chicago, Ill, USA) was used for statistical analysis. Simple descriptive analysis was done using median [IQR] or frequency (%).

To compare continuous variables in each sample, we used Wilcoxon signed-rank test. Sensitivity, specificity, positive predictive value (PPV), and negative predictive value (NPV) of the qualitative kit were compared to the gold standard GC. A *P*-value less than 0.05 was statistically significant.

## Results

Due to creamy nature and low quantity of some samples, five samples were eliminated from the study and 195 samples were tested. The measured ethanol, methanol, and formaldehyde contents of the samples are presented in Table 1.

**Table 1:** Results of the measurements by different methods in Indian/Iranian samples.

	**Median [IQR] Iranian samples (min, max) (*n* = 100)**	**Median [IQR] Indian samples (min, max) (*n* = 95)**	***P*-value**	**Median [IQR] total (min, max) (*n* = 195)**
Ethanol (v/v%) (*n* = 195)	24.55 [8.93, 39.50] (1.4, 57.8)	39.40 [27.50, 43.50] (7.3, 52.9)	>0.001	33.6 [22.10, 43.10] (1.4, 57.8)]
Methanol (mg L^−1^) (*n* = 195)	4,101.50 [467.50, 9,687.75] (0, 83,132)	26 [6, 74] (0, 644)	>0.001	118 [22, 4,290] (0, 83,132)
Ethanol/methanol ratio^*^	5.73 [1.70, 42.48] (0.05, 57,800.0)	1,228.12 [441.51, 6,933.33] (61.33, 50,100.0)	>0.001	216.2 [5.4, 1,821.4] (0.05, 57,800.0)
Formaldehyde (mg L^−1^) (*n* = 34)^†^	456 [185, 556] (0, 1,242)	1,927 [321, 2,200] (112, 2,742)	0.002	571 [228, 1,997.8] (0, 2,742)
Methanol kit (quantitative) (mg L^−1^) (*n* = 195)	4,375 [0, 11,322] (0, 84,960)	0 [0, 0] (0, 3,542)	>0.001	0 [0, 4,671.2] (0, 84,960)

* It is multiplied in 1,000 to have the same unit (mg L^−1^) and indicates the possibility of poisoning if this is less than 10. Higher values generally signify an apparently safer alcoholic beverage regarding potential for methanol poisoning. We added 1 mg L^−1^ to methanol free alcoholic beverages to calculate the ratio.

† Subject to missing data.

### GC vs. qualitative kit

A Wilcoxon signed-rank test showed that measuring methanol level yielded similar results using GC and quantitative new kit (*Z* = −0.328, p = 0.743). The median [IQR] methanol level was 118 [22, 4,290] (range; 0–83,132) mg L^−1^ by GC compared to 0 [0, 4,671] (range; 0–84,960) mg L^−1^ by the quantitative method.

### GC vs. quantitative kit

Table 2 shows the risk of methanol poisoning by drinking the beverage sample determined by GC and qualitative kit. The Kit was able to detect all safe and methanol-contaminated samples (110 and 60 cases respectively, 100% sensitivity).

**Table 2:** Sensitivity, specificity, positive and negative predictive values of the qualitative tests compared to the gold standard.

	**Sensitivity**	**Specificity**	**PPV**	**NPV**	**Accuracy**
Qualitative test for methanol	100 (94.04, 100)	81.48 (73.89, 87.64)	70.59 (62.75, 77.37)	100	87.18 (81.66, 91.53)
Added value of formaldehyde	100 (95.65, 100)	98.21 (93.70, 99.78)	97.65 (91.31, 99.39)	100	98.97 (96.34, 99.88)

### False positive samples and formaldehyde analysis

There were 25 samples with negative results when checked by GC analysis although they were reported positive using the qualitative kit. Formaldehyde measurement by UV/Vis analysis showed formaldehyde presence in 23 (92%) with a median [IQR] of 912 [249, 2,109] mg L^−1^ (range; 112–2,742). Adding the value of possible toxicity by formaldehyde in alcoholic beverage samples, the new calculation is presented in Table 2.

## Discussion

Studies investigating the potential of methanol poisoning of an alcoholic drink are scarce. Little has been published to date in the way of rapid or accessible methodology to evaluate for toxic alcohol levels in beverages. They usually rely on U.S. Bureau of Alcohol, Tobacco and Firearms (ATF), or general EU limit (33, 34).

Based on the EU definition, if methanol level of alcoholic beverages exceeds to 0.4 gram L^−1^ (or 4,000 mg L^−1^) of 40% v/v (or 400,000 mg L^−1^) ethanol solution or 10 gram L^−1^ (or 10,000 mg L^−1^) of absolute ethanol, the normal serum methanol level would be increased to more than 4 mg L^−1^ (upper normal value of methanol in a fasting person). This is not a “safe” dose and can result in chronic methanol poisoning. If an average adult person with 75-kg weight and about 41 liter of body water (55% of the total body weight) consumes 275 mL of a solution with the concentration of 30,000 mg L^−1^, it is sufficiently dangerous to advocate active treatment by causing a serum methanol level of 200 mg L^−1^ (acute methanol poisoning).

Ingestion of high levels of methanol contents of the non-standard alcoholic beverages can result in methanol poisoning. One major problem in the quality control process of alcoholic beverages production is the lack of accessible and cheap methods of methanol detection. This is especially important in the process of production of homemade alcoholic beverages in countries where ethanol use is banned.

Although CA is an easy and accurate method recommended by AOAC for this purpose, this reference method is difficult to carry out and is originally designed to measure formaldehyde. Further it reacts with both methanol and formaldehyde in solutions containing both (2, 5, 6, 15, 19). Thus, we used two qualitative and quantitative kits recently produced based on the CA method by an Iranian company to detect and measure formaldehyde and methanol contents of 195 original, handmade, and artificially-made alcoholic samples. To obtain a better interpretation of the results, a modified CA method was applied to measure formaldehyde contents of the suspected samples. These kits are free of the difficulties associated with traditional methods and their ease of application has been proven in previous studies (4, 5, 7).

Although both qualitative and quantitative kits are designed based on CA methods, the application and settings are for different purposes. The main differences of kits are shown in Table 3.

**Table 3:** Qualitative and quantitative advantages/disadvantages of CA kits.

**Qualitative**	**Quantitative**
For public before using alcoholic drinks with minimum literacy, average sensitivity and precision	Laboratories in lack of GC facilities, high sensitivity and precision
Determination of possibility of methanol poisoning, comparing methanol/ethanol ratio (EU standard)	Independent determination of methanol concentration
A rapid test in 5 min using	A laboratory test in 20 min using calorimetric machines
Limit of Detection (LOD) 4,000 mg/l^−1^	Limit of Detection (LOD) 700 mg/l^−1^
No limit of Quantification (LOQ)	Limit of Quantification (LOQ) 1,500 mg/l^−1^

Although the ethanol level of each sample was measured only by the GC method, the methanol content of the samples were evaluated using both GC and quantitative/qualitative kits. The permitted dose of methanol was used to better interpret and compare GC and qualitative kit results. The results of the quantitative and qualitative kits served as confirmation of each other in all cases. However, the results of the kits were different from GC in some samples, this could be due to the presence of formaldehyde, considering the CA method mechanism. This should be considered as an advantage using simultaneous evaluation of methanol and formaldehyde in samples, from our point of view (Table 3).

The quantitative kit is unable to detect concentrations less than 1,500 mg L^−1^ (LOQ). For this reason the median [IQR] value was zero if it was less than the detection limit compared to GC measurement. In other words, all measured values less than this amount by the quantitative kit were considered zero in the comparative calculations with GC.

Although some samples lacked methanol and formaldehyde, others had only methanol or both. Formaldehyde content of the samples was measured only for better interpretation of the qualitative kit results compared to the GC. Contrary to expectations and GC results, some tests resulted in a positive result for methanol using these kits.

Evaluation of three Indian samples was impossible due to their creamy consistency. Almost all samples had different concentrations of methanol below the permitted concentration (EU standard), compatible with the previous studies (4, 5, 7).

If the samples were reported to be positive using the qualitative kit but GC showed negative results, they were considered suspicious samples. We have shown that this issue is mainly due to the presence of formaldehyde, particularly in Indian samples, which interferes with the function of the kit. Based on the previous reasoning with regards to interfering substances and the high possibility of presence of formaldehyde in these beverages, it was concluded that the differing results were due to the presence of formaldehyde. The presence of formaldehyde in alcoholic beverages may cause long-term toxicity. A kit that can detect this has an added advantage. Formaldehyde ranks second on the priority control list of toxic chemicals in China as a serious threat to human health (16).

Iranian and Indian samples may differ in terms of the source of alcohol production. The main source of sugar used in the production of alcoholic beverages in Iran is raisin. In India, however, sugarcane extract is mostly used for this purpose. In order to prepare raisin, grape must be exposed to sunlight for days necessitating a long process, and the sudden atmospheric changes can cause irreparable damage to the crop. Therefore, gardeners may use unauthorized herbicides for this purpose. Two–four dichlorophenoxy acetic acid (2,4-D) is a common, cheap, and effective herbicide and should not be essentially used for broadleaf plants such as grape (4, 35, 36). However, its low price and ease of access, as well as lack of proper monitoring of the food production methods have resulted in its use to dry grape in the vineyards. While this compound remains on the raisin it can cause a false positive results (4). Two out of 25 false positive samples had no formaldehyde in our analysis, which may be due to the presence of this herbicide, but more research is required to confirm this.

### Limitations

Lack of resources prevented further investigations on the possible existence of 2,4-D or other phenoxy agents in the samples.

We were not able to measure formaldehyde in all samples and only measured formaldehyde in false positive samples; there might be contamination with formaldehyde and methanol in other samples not detected in the current study.

## Conclusion

Using the new kit, we can assess the safety of alcoholic beverages before consumption using both qualitative and quantitative kits. Such detection methods may prevent methanol toxicity and even outbreaks if available for use in the community. Although positive results may indicate the risk of acute or chronic methanol toxicity from the beverage, it may also indicate the risk of chronic toxicity by formaldehyde. In countries like India and Iran where much harm is seen due to methanol, and the burden of this on the public health system, these kits are able to detect the potential for toxicity and identify safer alcoholic beverages. Feasibility and low cost could be an invaluable public health measure to reduce associated harms.

## Data availability statement

The original contributions presented in the study are included in the article/supplementary material, further inquiries can be directed to the corresponding author/s.

## Author contributions

AB, AR, and HH-M participated in the study design. NS, KK, HH-M, and AR participated in the acquisition and interpretation of all data. NZ wrote the first draft of manuscript. NZ, AB, AR, and HH-M critically revised the manuscript. All the authors have read the journal's authorship agreement and the manuscript has been reviewed by and approved by all named authors.

## Funding

This study was supported by Shahid Beheshti University of Medical Sciences (Grant no. 17153).

## Conflict of interest

The authors declare that the research was conducted in the absence of any commercial or financial relationships that could be construed as a potential conflict of interest.

## Publisher's note

All claims expressed in this article are solely those of the authors and do not necessarily represent those of their affiliated organizations, or those of the publisher, the editors and the reviewers. Any product that may be evaluated in this article, or claim that may be made by its manufacturer, is not guaranteed or endorsed by the publisher.

## Abbreviations

2: 4-D: 2–4 dichlorophenoxyacetic acid
AOAC: Association of Official Analytical Chemists
CA: Chromotropic acid
Mn: Manganese
EtOH: Ehanol
FID: Flame Ionization Detector
FT-IR: Fourier-transform infrared spectroscopy
GC: Gas Chromatography
GC–MS: Gas Chromatography-Mass Spectrometry HPLC: High-performance liquid chromatography
IARC: International Agency for Research on Cancer
IQR: Inter Quartile Range
NIOSH: National Institute for Occupational Safety and Health
NPV: Negative Predictive Value
PPV: Positive Predictive Value
SD: Standard Deviation
SPSS: Statistical Package for the Social Sciences
WHO: World Health Organization.
